# Stratification of cancer and diabetes based on circulating levels of formate and glucose

**DOI:** 10.1186/s40170-019-0195-x

**Published:** 2019-04-24

**Authors:** Matthias Pietzke, Salvador Fernandez Arroyo, David Sumpton, Gillian M. Mackay, Begoña Martin-Castillo, Jordi Camps, Jorge Joven, Javier A. Menendez, Alexei Vazquez, On behalf of the METTEN study group, Sonia Pernas, Joan Dorca, Isabel Álvarez-López, Susana Martínez, Jose Manuel Pérez-García, Norberto Batista López, César A. Rodríguez-Sánchez, Kepa Amillano, Severina Domínguez Fernández, Maria Luque-Cabal, Idoia Morilla, Gemma Viñas, Javier Cortés, Begoña Martin-Castillo, Javier A. Menendez

**Affiliations:** 1 Cancer Research UK Beatson Institute, Switchback Road, Bearsden, Glasgow, G61 1BD UK; 2 Unitat de Recerca Biomèdica, Hospital Universitari de Sant Joan, IISPV, Rovira i Virgili University, Reus, Spain; 3 Unit of Clinical Research, Catalan Institute of Oncology, Girona, Spain; 4 ProCURE (Program Against Cancer Therapeutic Resistance), Metabolism & Cancer Group, Catalan Institute of Oncology, Girona, Catalonia Spain; 5 Girona Biomedical Research Institute (IDIBGI), Girona, Spain; 6 Institute of Cancer Sciences, University of Glasgow, Glasgow, UK

**Keywords:** Cancer, Obesity, Biomarker, Serum metabolomics, Formate

## Abstract

**Background:**

Serum and urine metabolites have been investigated for their use as cancer biomarkers. The specificity of candidate metabolites can be limited by the impact of other disorders on metabolite levels. In particular, the increasing incidence of obesity could become a significant confounding factor.

**Methods:**

Here we developed a multinomial classifier for the stratification of cancer, obesity and healthy phenotypes based on circulating glucose and formate levels. We quantified the classifier performance from the retrospective analysis of samples from breast cancer, lung cancer, obese individuals and healthy controls.

**Results:**

We discovered that circulating formate levels are significantly lower in breast and lung cancer patients than in healthy controls. However, the performance of a cancer classifier based on formate levels alone is limited because obese patients also have low serum formate levels. By introducing a multinomial classifier based on circulating glucose and formate levels, we were able to improve the classifier performance, reaching a true positive rate of 79% with a false positive rate of 8%.

**Conclusions:**

Circulating formate is reduced in HER2+ breast cancer, non-small cell lung cancer and highly obese patients relative to healthy controls. Further studies are required to determine the relevance of these observations in other cancer types and diseases.

**Electronic supplementary material:**

The online version of this article (10.1186/s40170-019-0195-x) contains supplementary material, which is available to authorized users.

## Background

Serum and urine biomarkers can enable the widespread deployment of disease screening. A successful example is the use of fasting serum glucose levels to diagnose diabetes [1]. In the context of cancer, several studies have been conducted with the aim of identifying serum or urine metabolites that could distinguish cancer patients from healthy controls [2–5]. Within the range of metabolites analysed in previous studies, no single metabolite alone can be used to discriminate between samples from cancer patients and healthy controls in a reliable manner. Instead, complex metabolites signatures are devised. The general consensus from these studies is that a cancer diagnostic test based on a single metabolite, mechanistically linked to cancer metabolism, is not feasible.

Yet, we have previously observed that tumour-bearing mice have high serum formate levels relative to matched controls [6]. We therefore hypothesised that formate levels could be utilised to screen for cancer disease in the human population. To test this hypothesis, we performed metabolite analysis of serum/plasma samples from a Spanish cohort of breast cancer patients, lung cancer patients, obesity patients and healthy controls. In contrast to our observations in mice, circulating formate levels are significantly lower in cancer patients than in healthy controls. Formate levels were also found significantly lower in patients with obesity, forcing us to concomitantly stratify obesity and cancer patients from healthy controls. By introducing a multinomial classifier based on glucose and formate levels, we were able to improve the classifier performance, reaching a true positive rate of 79% with a false positive rate of 8%.

## Methods

### Participants

We included plasma samples from 80 patients with severe obesity (i.e., body mass index [BMI] > 40 kg/m^2^) enrolled in an on-going study aimed to establish the prevalence of non-alcoholic steatohepatitis (NASH). Patients were categorised according to the presence (*n* = 45) or absence (*n* = 35) of type 2 diabetes mellitus (T2DM) as defined by levels of fasting plasma glucose > 7.0 mmol/L and HbA_1c_ > 48 mmol/mol (6.5%). Patients were excluded if aged < 25 years and self-reported alcohol consumption was higher than 25 g/day or conflicted with the assessment by relatives. Other exclusion criteria included positive values in markers indicative of autoimmune hepatitis, hepatitis B or hepatitis C, and patients with a history of cardiac disease, liver disease of non-metabolic aetiology, current infections, chronic inflammatory diseases or cancer. For comparisons, we used bio-banked samples (*n* = 50) of healthy non-obese controls from a previous, unrelated population study. Non-alcoholic fatty liver disease (NAFLD) and T2DM were discarded via ultrasound and laboratory data obtained in health checkups, that is, non-NAFLD, non-diabetic controls, using a population-based approach.

We prospectively collected fasting plasma samples (*n* = 58) of patients (84% male) with unresectable locally advanced non-small cell lung cancer before chemoradiotherapy. Patients were excluded if they presented with metastatic disease or previous oncologic intervention. All patients underwent staging with PET/CT imaging, IIIA or IIIB in a 50/50 proportion. Mediastinum staging also required endobronchial ultrasound or a mediastinoscopy approach in a significant number of patients. All patients had a brain assessment by MRI. Tumour histology revealed adenocarcinoma in 31 patients and squamous cell carcinoma in 20. Before treatment, all patients had an excellent (0–1) ECOG-PS score (Eastern Cooperative Oncology Group performance status). T2DM was present in 16 patients, hypertension in 19, moderate-to-high consumption of alcohol in 31%, and 50% were current smokers. Written informed consent was obtained from all participants as required by the ethics committee of the Hospital Universitari Sant Joan de Reus (Reus, Spain).

We also prospectively collected serum samples (*n* = 68) from patients with early, non-metastatic HER2-positive breast cancer that was recruited into the METTEN study (EU Clinical Trials Register, EudraCT number 2011-000490-30; registered on 28 February 2011, https://www.clinicaltrialsregister.eu/ctr-search/trial/2011-000490-30/ES) [7]. Patients were eligible if they met the following criteria: previously untreated, operable, locally advanced, inflammatory breast cancer > 2.0 cm in the largest clinical diameter and confirmed HER2 positivity (either immunohistochemistry 3+ or 2+ and positive for fluorescent or chromogenic in situ hybridization). Other inclusion criteria were age 18–75 years, baseline ECOG-PS score of 0 or 1 and baseline left ventricular ejection fraction ≥ 50% measured by echocardiography or multiple gated acquisition scan; normal organ and bone marrow function (absolute neutrophil count ≥ 1500/μL, platelets ≥ 100,000/μL, total bilirubin ≤ 1.5× the upper limit of normal [ULN], serum creatinine ≤ 1.5× ULN, AST and ALT ≤ 2.5× ULN); ability to swallow and retain oral medication and blood glucose levels ≥ 70 mg/dL (3.9 mmol/L). Patients were excluded from this study if they had impaired cardiac function (e.g. uncontrolled or symptomatic angina, clinically significant arrhythmias, congestive heart failure, transmural myocardial infarction); uncontrolled hypertension; concurrent treatment with therapies that can alter insulin levels (including chronic treatment with oral corticoids); and metabolic disease (e.g., T1/2 DM, obesity [BMI > 30 kg/m^2^]; impaired glucose tolerance [> 128 mg/dL], hypercholesterolaemia or hypertriglyceridaemia of grade ≥ 3 according to CTC-NCIC version 4.0). Other exclusion criteria were metastatic disease; bilateral breast cancer; any prior treatment for breast cancer; other malignancies or less than 10 years from prior malignancies (except curatively treated basal cell carcinoma, squamous cell carcinoma of the skin or carcinoma in situ of the cervix); inadequate renal function (creatinine clearance < 60 mL/min); impaired liver function; enolism (average consumption of 3 alcoholic beverages/day); significant dementia; altered mental status (or any psychiatric condition that would prohibit the understanding or rendering of informed consent); pregnancy; and lactation. The ethics committee of the Dr. Josep Trueta Hospital (Girona, Spain) and independent Institutional Review Boards at each site participating in the METTEN study approved the protocol and any amendments. All procedures were in accordance with the ethical standards of the institutional research committees and with the 1964 Helsinki Declaration and its later amendments or comparable ethical standards. Informed consent was obtained from all individual participants included in the METTEN study.

In all participants, venous blood was collected, after an overnight fast, into sodium EDTA-containing tubes (plasma) or into tubes with no anticoagulants added (serum). The tubes were centrifuged at 2500×*g* at 4 °C, and plasma or serum was stored at − 80 °C until used to minimise preanalytical errors.

### Formate quantification

Formate was quantified by gas chromatography–mass spectrometry (GC-MS) (Agilent) as described previously [8]. Briefly, 40 μL of the samples were mixed with 20 μL of internal standard (d2-formate, 50 μM), 10 μL of NaOH (1 N), 50 μL of pyridine and 5 μL of benzyl alcohol. Derivatisation was performed by adding 20 μL of methyl chloroformate while vortexing. After addition of 100 μL of methyl tertiary butyl ether and 200 μL H_2_O followed by vortexing for 10 s and centrifugation (10 min at max *g*), the apolar phase was transferred to a GC-vial and capped. Blank samples (water) and formate standards with known concentration were prepared in a similar manner and measured with the samples to subtract background and validate the quantification. Peak areas for formate and d_2_-formate were extracted and processed with MassHunter Quantitative analysis software (version B.06.00—Agilent Technologies). Quantification was performed by comparing the peak area of formate (*m*/*z* of 136) against that of d_2_-formate (*m*/*z* = 138) after correcting for background signals. Because of the high number of samples, we measured them in blocks, but each block contained every sample type and samples were randomised within each block. Each block of samples also included reference samples that were used as quality controls. Additional file 1: Figure S1 shows the measured formate values for these samples relative to the spiked labelled formate in those references. The data demonstrates the good quality of the formate quantification in the range 0–100 μM formate, which corresponds to the readouts observed in human serum samples.

### Targeted liquid chromatography–mass spectrometry quantification

Other metabolites were measured as previously described [9], with the extraction slightly modified to prevent clogging of the column. Both plasma and serum samples were diluted 1:100 in extraction solution (methanol to acetonitrile to water [5:3:1 *v*/*v*]), followed by a vortexing step and 10 min shaking at 4 °C. Samples were then centrifuged (max *g*, 10 min, 4 °C), transferred to a new Eppendorf tube and stored overnight at − 80 °C. After defrosting for 10 min on ice, samples were centrifuged a second time (max *g*, 10 min, 4 °C), transferred to liquid chromatography (LC)-vials, separated on a ZIC-pHILIC column and analysed with a Q-Exactive-orbitrap MS (Thermo Fisher). As a quality control for the LC-mass spectrometry (LC-MS) quantification, we spiked in a ^13^C- and ^15^N-amino acid mixture. (Additional file 1). The quantified amino acid concentrations were found in a range previously reported for human serum [10] (Additional file 1: Table S2 and Figure S2). Using amino acids as quality controls, we identified matrix effects due to co-elution with EDTA in all plasma samples (Additional file 1: Figures S3–S6).

### Relative mutual information

The mutual information of a classifications system S* relative to a reference system *S* is calculated as

$$ I\left({S}^{\ast },S|U\right)={\sum}_{\mathrm{ab}}{p}_{\mathrm{ab}}\ln \left(\frac{p_{\mathrm{ab}}}{q_a{r}_b}\right), $$

where *U* is the set of all samples considered in the study, *p*_ab_ is the fraction of individuals that belong to class *a* and *b* in the classification systems *S* and S*, respectively, *q*_*a*_ is the fraction of individuals that belong to class *a* in the classification system *S* and *r*_*b*_ is the fraction of individuals that belong to class *b* in the classification system S*. The relative mutual information is defined here as the mutual information normalised to its maximum attainable value when S* = *S*,

$$ i\left({S}^{\ast },S|U\right)=\frac{I\left({S}^{\ast },S|U\right)}{-{\sum}_a{q}_a\ln {q}_a}. $$

### Cross-validation

We consider the sets *H*, *C* and *O* as containing the samples of healthy controls, cancer patients and obesity patients, respectively, as provided in the reference annotation. We also consider the sets H*, C* and O* as containing the samples imputed as healthy controls, cancer patients and obesity patients, respectively, based on a given classifier. As indicated above, *U* is the set of all samples. A training set (*T* ⊂ *U*) or a validation set (*V* ⊂ *U*) are also defined depending on whether we are performing a receiver operating characteristic (ROC) study or a cross-validation analysis, as described below. The training set is used to determine the optimal parameters of the classifier. The validation set is used to quantify the TPR and FPR according to the equations

$$ {\mathrm{TPR}}_C=\frac{\left|V\cap C\cap {C}^{\ast}\right|}{\left|V\cap C\right|}, $$

$$ {\mathrm{FPR}}_C=\frac{\left|V\cap H\cap {C}^{\ast}\right|+x\left|V\cap O\cap {C}^{\ast}\right|}{\left|V\cap H\right|+x\left|V\cap O\right|}, $$

$$ x={p}_O\frac{\left|V\cap H\right|}{\left|V\cap O\right|} $$

where *X*∩*Y* denotes the intersection between *X* and *Y* (elements common to *X* and *Y*), |*X*| denotes the size of *X* (number of elements in *X*), and *p*_*O*_ is the obesity prevalence in the population. The generalisation of this equation for more than two diseases is straightforward and reported in the Additional file 1.

### Receiver operating characteristic plots

In this case, both the training and validation sets contain all samples (*T*=*V*=*U*). The ROC plots were generated by changing *F*_*T*_ or (*G*_*T*_,*F*_*T*_) across all observed values. For each threshold, we classified every sample in the validation set and determined the true and false positive rate (TPR and FPR, respectively).

### Cross-validation

For each realisation of the cross-validation procedure, each sample in the study was assigned to a training set (*T*) with probability 0.75 or to a validation set (*V*) otherwise. Results were averaged over 100,000 realisations of (*T*,*V*). For each quantity of interest, the values separating the 5, 50 (median) and 95% lower values from the remaining higher values were calculated. The results are then reported as median (5–95% values).

### *F*-classifier validated in *H* + *C*

The imputed classes (S*) are determined as

$$ {S}_i^{\ast}\left({F}_T\right)=\left\{\begin{array}{l}c\kern1em if\;{F}_i<{F}_T\\ {}h\kern1em Otherwise\end{array}\right. $$

where *F*_*i*_ denotes the serum formate level of sample *i* and *F*_*T*_ is a predefined threshold. The best formate threshold was calculated as

*F*_0_ = arg max *i*(*S*, *S*^∗^(*F*_*T*_)|*V* ∩ (*H* ∪ *C*))

C* and H* were defined as the set of all samples imputed by the *F*_0_-classifier as having cancer or being healthy, respectively. The validation is conducted setting the obesity prevalence to zero (*p*_*O*_ = 0). In this case, the obesity class is irrelevant.

### *F*-classifier validated in *H* + *C* + *O*

Proceeds as described above, but the obesity prevalence is set to 20% (*p*_*O*_ = 0.2).

### (*G*,*F*)-classifier validated in *H* + *C* + *O*

The imputed classes (S*) are determined as

$$ {S}_i^{\ast}\left({G}_T,{F}_T\right)=\left\{\begin{array}{l}o\kern1em \mathrm{if}\;{G}_i>{G}_T\\ {}c\kern1em \mathrm{if}\;{G}_i<{G}_T\;\mathrm{and}\;{F}_i<{F}_T\\ {}h\kern1em \mathrm{Otherwise}\end{array}\right. $$

where *G*_*i*_ denotes the serum glucose level of sample *i* and *G*_*T*_ is a predefined threshold. The best formate threshold was calculated as

$$ \left({G}_0,{F}_0\right)=\underset{\left({G}_T,{F}_T\right)}{\arg \max }i\left(S,{S}^{\ast}\left({G}_T,{F}_T\right)|V\cap \left(H\cup C\cup O\right)\operatorname{}\right) $$

where C*, O* and H* were defined as the set of all samples imputed by the (*G*_0_,*F*_0_)-classifier as cancer, obesity or healthy, respectively. The obesity prevalence is set to 20% (*p*_*O*_ = 0.2).

## Results

**Fig. 1:**
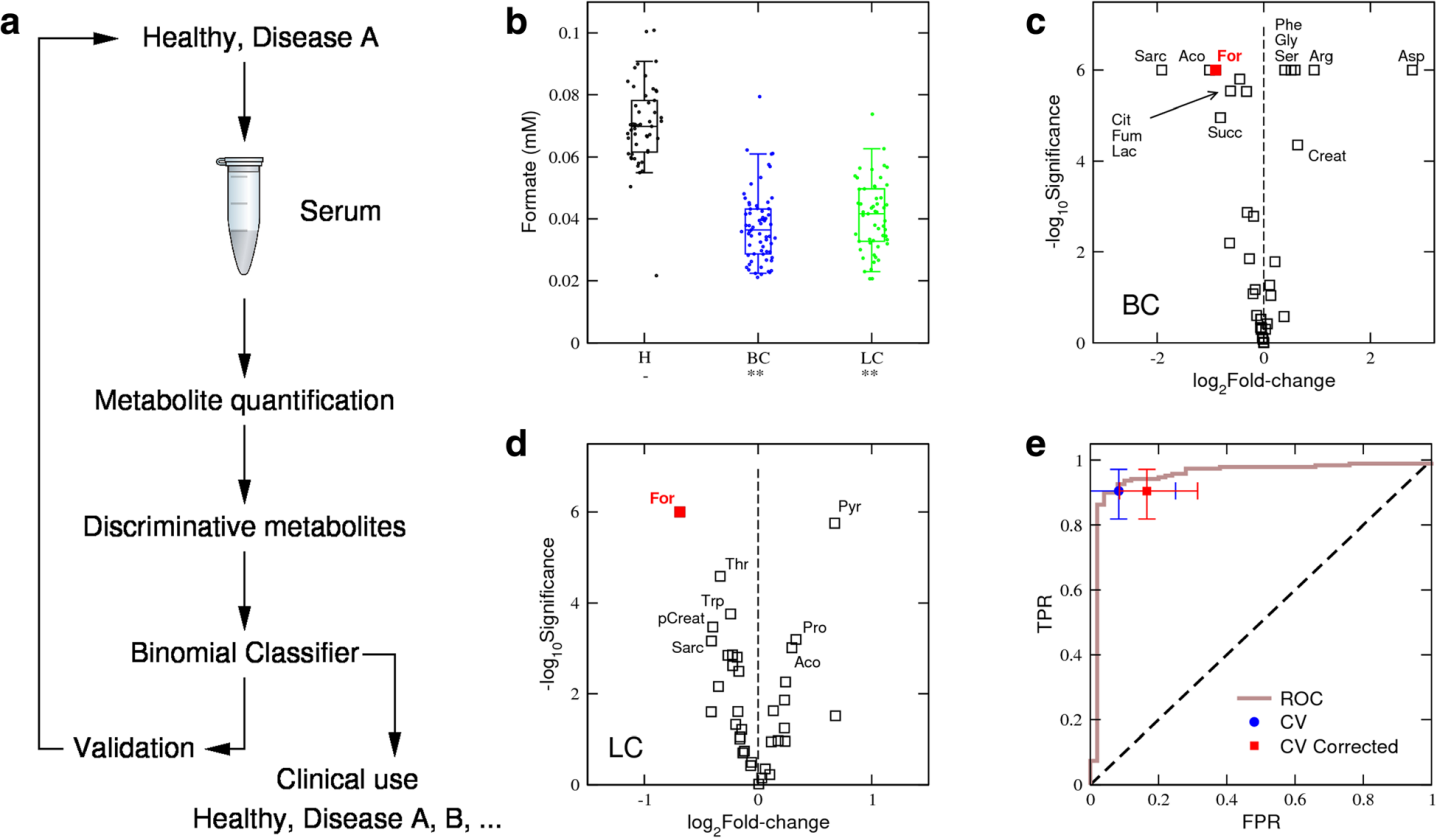
Formate classifier. **a** Schematic representation of a standard case-control study to identify and validate disease metabolite biomarkers. **b** Box plots of serum formate levels across healthy controls (H), HER2+ breast cancer (BC) and non-small cell lung cancer (LC) samples. Each point represents a sample, the error bars indicate the range excluding the lowest 5% and highest 5% values, boxes the range excluding the lowest 25% and highest 25% values, and the line within the box the median. Asterisk/double asterisks denote a significant difference of 10^−3^/10^−6^ relative to healthy controls (−), two-tailed *t* test with unequal variance. **c**, **d** Volcano plots reporting the statistical significances vs fold change of metabolite levels relative to the healthy controls, in BC (**c**) and LC (**d**). Each point represents a metabolite and selected metabolites are indicated by their abbreviated name. **e** ROC plot for the formate-based classifier (brown line), together with the FPR and TPR obtained from cross-validations not corrected (CV) and accounting (CV corrected) for obesity incidence The symbol reports the median and the error bars the observed range excluding the 5% lowest and largest values

Our goal was to develop a classifier that differentiates cancer and healthy samples based on circulating metabolite levels. We followed a standard case-control study where biological samples were obtained from patients with a given disease and from healthy controls (Fig. 1a). The disease of interest was cancer and the biological samples were serum from women with HER2/Erb2-positive primary breast cancer, plasma from non-small cell lung cancer patients and plasma from healthy controls (Table 1). Mechanistic studies (e.g. animal models) can inform the choice of metabolites to be screened; a good example is blood glucose in the context of diabetes [1]. In the context of cancer, we previously reported that tumour-bearing mice have high serum levels of formate relative to matched controls [6]. We therefore hypothesised that formate levels could be used to screen for cancer in the human population.

**Table 1:** Characteristics of the study population

	H	BC	LC	OD−	OD+
	*n = 50*	*n = 68*	*n = 56*	*n = 46*	*n = 35*
Age	48 ± 14	48 ± 11	66 ± 9	50 ± 10	42 ± 12
BMI	27 ± 6	25 ± 3	27 ± 5.1	46 ± 7	51 ± 9

*H* healthy controls, *BC* early stage non-metastatic HER2+ breast cancer patients before treatment, *LC* unresectable locally advanced non-small cell lung cancer patients before chemoradiotherapy, *OD*− severe obesity patients without T2DM, *OD*+ severe obesity patients with T2DM. Values are reported as mean ± SD

We quantified circulating formate in the biological samples using a gas chromatography–mass spectrometry (GC-MS) protocol [8]. In contrast to our previous observations in mice, circulating formate levels were significantly lower in breast and lung cancer patients than in healthy controls (Fig. 1b). To compare the discriminative power of formate relative to that of other metabolites, we quantified the levels of a broad spectrum of metabolites using LC-MS. Among the metabolites quantified, formate showed the highest fold reduction in breast and lung cancer relative to healthy control samples (Fig. 1c, d and Additional file 1: Table S1). We found only three metabolites with a consistent and significant change between each cancer type and healthy controls: formate, glutamate and sarcosine. The fold change of glutamate was, however, small compared with that observed for formate. Sarcosine exhibited a fold change similar to that of formate, but its levels were highly correlated with those of formate (Pearson correlation 0.60, *p* = 10^−6^, permutations test).

### *F*-classifier

These observations encouraged us to develop a cancer classifier using circulating formate levels as input. Specifically, samples were imputed as cancer if formate levels were below a predefined threshold (*F*_*T*_) and as healthy (or no cancer) otherwise. Changing the formate threshold, we obtained an excellent ROC curve (Fig. 1e), with a TPR close to 100% almost independently of the FPR. The classifier also performed well in a cross-validation analysis, where 75% of the cancer and healthy samples were used to estimate the best *F*_*T*_ and the remaining 25% of samples were used for validation (Fig. 1e, blue circle). Taking an average of over 100,000 cross-validations, we obtained a TPR of 90% (82–97%) and an FPR of 8% (0–25%). Based on this standard case-control analysis, we conclude that formate alone can be used to screen for cancer in the human population.

**Fig. 2:**
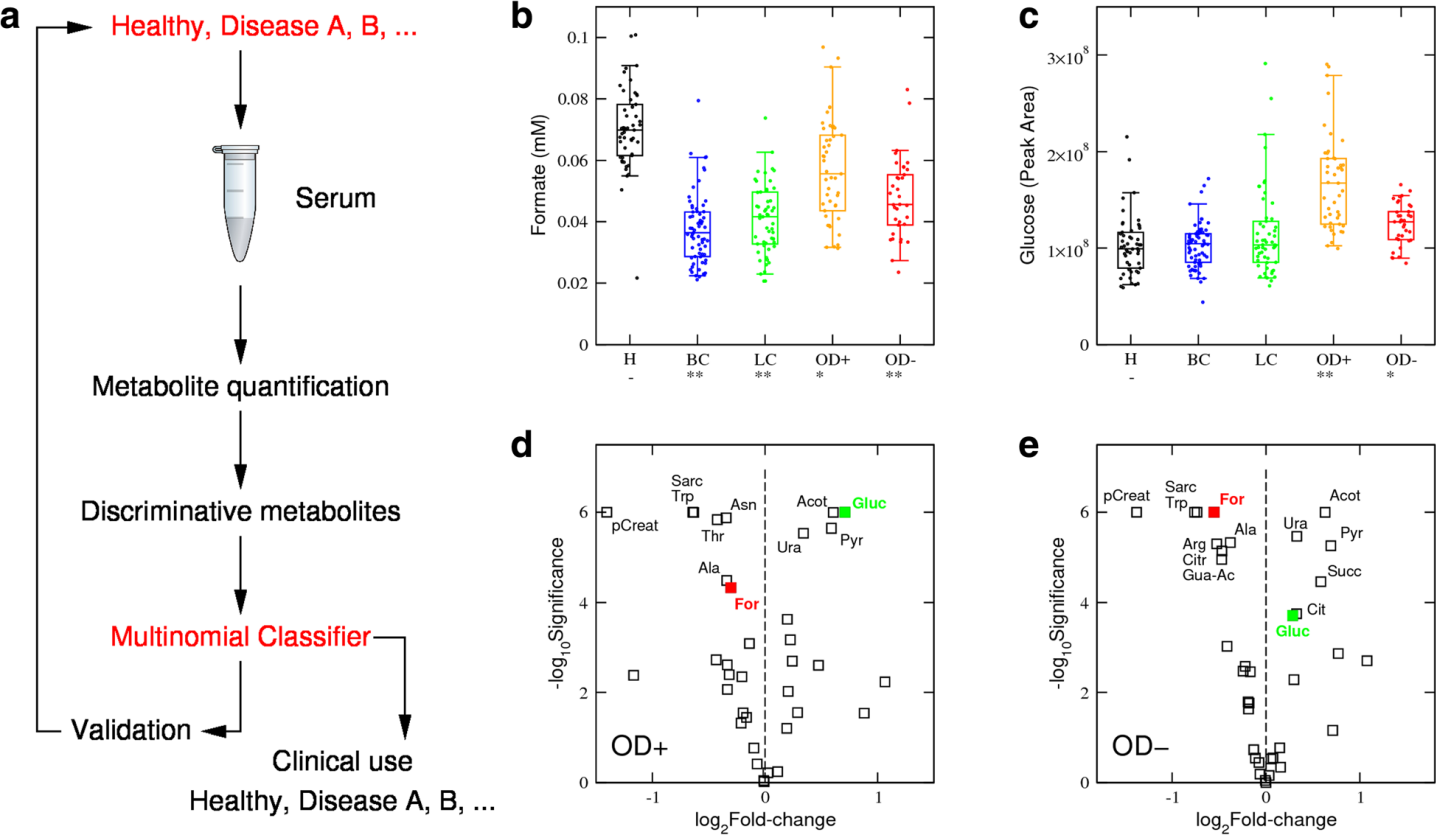
(Glucose,Formate) classifier. **a** Schematic representation of the proposed study design to identify and validate disease metabolite biomarkers considering the prevalence of multiple diseases. Box plots of formate (**b**) and glucose (**c**) levels across the healthy controls (H), HER2+ breast cancer (BC), non-small cell lung cancer (LC), severe obesity without diabetes (OD−) and severe obesity with diabetes (OD+) samples. Each point represents a sample, the error bars indicate the range excluding the lowest 5% and highest 5% values, boxes the range excluding the lowest 25% and highest 25% values, and the line within the box the median. Asterisk/double asterisks denote a significant difference of 10^−3^/10^−6^ relative to healthy controls (−), two-tailed *t* test with unequal variance. **d**, **e** Volcano plots reporting the statistical significances vs fold change of metabolite levels in the indicated group relative to the healthy controls, in OD+ (**d**) and OD- (**e**). Each point represents a metabolite and selected metabolites are indicated by the labels

In the clinical setting, we would encounter patients with cancer and healthy controls and individuals with other underlying diseases as well. Of particular relevance is obesity, which is estimated prevalence at 11–15% globally and is projected to reach 20% in 2025 [11]. To investigate the impact of obesity in the performance of the formate-based classifier, we expanded the cohort to include plasma samples from patients with obesity (Table 1) and the levels of formate and other metabolites were quantified. We then repeated the cross-validation analysis including obesity samples in the validation cohort, at a rate of 20% per healthy control (Fig. 1e, red square). With the inclusion of obesity in the validation cohort, the FPR increased significantly to 17% (9–31%) (Fig. 1e, red square vs blue circle, *p* = 10^−5^, Welch test). These observations illustrate how a biomarker may seem to perform quite well in a single disease (e.g. cancer) and healthy control study. Yet, the classifier’s performance deteriorates when tested in the human population, due to the prevalence of other diseases affecting the biomarker (e.g. obesity). To overcome this caveat, we transformed the study design from a single disease and healthy controls (Fig. 1a) to multiple diseases and healthy controls (Fig. 2a). The key development is a validation set representing the relevant diseases that may be encountered in the human population. By relevant, we mean those diseases with similar biomarker profiles that may confound the discrimination between them. This also entails a change in the methodology from a binomial classifier (positive or negative) to a multinomial classifier (disease A, disease B,…, healthy).

To demonstrate the feasibility of this multinomial approach, we used the simultaneous diagnosis of cancer and obesity as a case study. In obesity, patient formate levels span a range from those observed in healthy individuals to those seen in cancer patients (Fig. 2b), which limits the use of a classifier for cancer based solely on formate levels. Indeed, a subset of patients with obesity presented formate levels as low as those observed in cancer patients. Accordingly, to address the issue of identifying cancer patients using serum formate, we have to discriminate additionally between cancer and obesity. In agreement with previous evidence [1], glucose levels are increased in patients with obesity relative to controls, independently of whether or not these patients have diabetes (Fig. 2c). As anticipated, glucose was among the metabolites with the highest fold increase in obesity patients with or without diabetes relative to healthy controls (Fig. 2d, e). By contrast, glucose levels were not significantly different between cancer patients and healthy controls (Fig. 2c).

### (*G*,*F*)-classifier

**Fig. 3:**
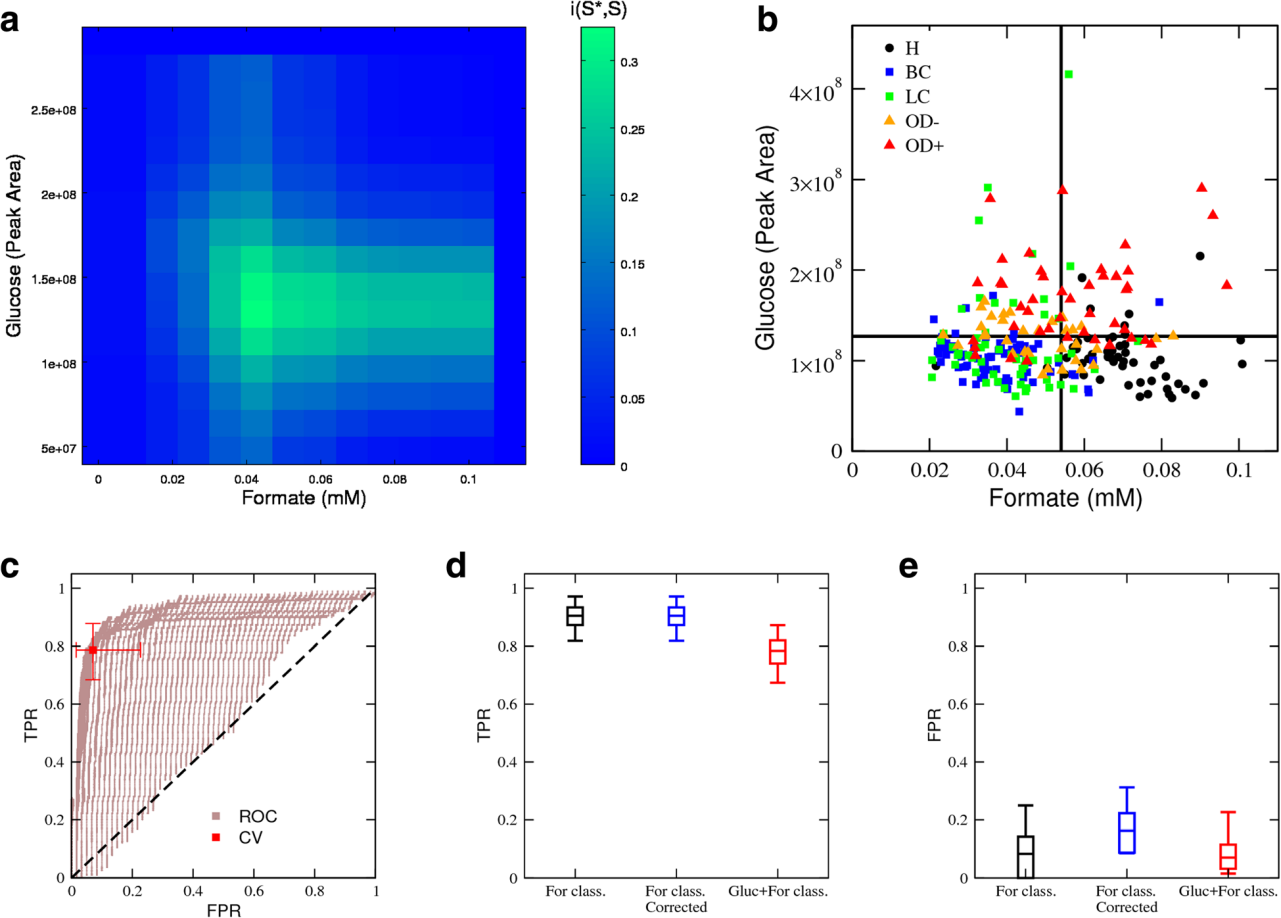
Performance of the (Glucose,Formate) classifier. **a** Colour map of the relative mutual information between the classifier prediction and the reference classification as a function of the formate and glucose thresholds. **b** Scatter plot of human serum samples as a function of the formate and glucose concentrations, colour coded by the sample subtypes. The horizontal and vertical lines represent the best glucose and formate threshold, respectively. **c** ROC plot for the glucose + formate-based classifier, focusing on the cancer class. The brown area reports the FPR and TPR for different formate and glucose thresholds and the red symbol the corresponding cross-validation values (symbol for median and error bars for observed range excluding the 5% lowest and largest values). **d**, **e** Comparison of the different classifiers according to their TPR (**d**) and FPR (**e**). Corrected indicates accounting for obesity incidence. Error bars indicate the range excluding the lowest 5% and highest 5% values, boxes the range excluding the lowest 25% and highest 25% values, and the line within the box the median

Based on these observations, we designed the following decision tree classifier. First, the samples were classified as *obesity* (glucose > *G*_*T*_) or *other* (glucose < *G*_*T*_), where *G*_*T*_ is a predefined glucose threshold. Subsequently, the *other* group was stratified based on formate levels into *cancer* (formate < *F*_*T*_) or *healthy* (formate > *F*_*T*_), where *F*_*T*_ is a predefined formate threshold. We first varied (*G*_*T*_,*F*_*T*_) over the range of observed glucose and formate levels. For a given (*G*_*T*_,*F*_*T*_), we classified all samples and determined the relative mutual information, *i*(S*,*S*), between the classifier status prediction (S*) and the actual status (*S*). The relative mutual information measures the similarity between two classification systems. *i*(S*,*S*) takes the maximum value 1 when the classifier has a perfect match with the actual status (S* = *S*) and is zero when the classifier predictions are uncorrelated from the actual status. Figure 3a shows *i*(S*,*S*) as a heatmap in the (*G*_*T*_,*F*_*T*_) plane. The highest relative mutual information is obtained for *G*_*T*_ = 1.3 × 10^8^ (peak area) and *F*_*T*_ = 0.054 mM, resulting in *i*(S*,*S*) = 0.36 (*p* = 10^−5^, permutation test, 10^5^ permutations). Figure 3b reports the distribution of the serum samples profiled with the (*G*_*T*_,*F*_*T*_), colour coded by status. The lines separate the (*G*_*T*_,*F*_*T*_) plane into different regions based on the optimal values of the aforementioned *G*_*T*_ and *F*_*T*_. From the visual inspection of this figure, it can be concluded that the classifier performs well at separating the different groups.

We next performed an ROC analysis of the (*G*_*T*_,*F*_*T*_) classifier to quantify its performance in terms of TPR and FPR. Given that we have two disease classes, cancer and obesity, we determined these quantities focusing either on cancer as the positive event and the remainder as negative, or on diabetes as the positive event and the remainder as negative. We note that the ROC plot associated with two or more biomarkers is generally a surface in the (FPR,TPR) plane (Fig. 3c). The ROC plot for cancer demonstrates that the cancer classifier can reach a TPR above 80% with an FPR below 20% (Fig. 3c). We also performed a cross-validation analysis where 75% of the samples were used for training and the remaining 25% samples were used for validation, after correcting for the obesity prevalence. The training set was used to determine the choice of (*G*_*T*_,*F*_*T*_) maximising *i*(S*,*S*) and the validation set was used to estimate the TPR and FPR. Taking an average of over 100,000 cross-validations, we obtained a TPR of 79% (63–92%) and an FPR of 23% (12–37%), for the cancer classifier (Fig. 3c, red square).

The performance of the classifiers discussed above is compared side-by-side in Figs. 3d, e. The TPR of the *F*-classifier was not affected by the inclusion of obesity patients in the validation cohort (Fig. 3d, blue vs black). By contrast, the FPR increased when the *F*-classifier is validated in a cohort including obesity patients, which is closer to what is expected in the clinical context (Fig. 3e, blue vs black). This increase in FPR is corrected by the (*G*,*F*) classifier, as a result of the inclusion of glucose as a biomarker to impute obesity (Fig. 3e, red vs black). This improvement comes at the expense of a reduction in the TPR (Fig. 3d, red vs black) because a small fraction of the cancer patients is imputed as belonging to the obesity class. These are the blue and green symbols above the horizontal line in Fig. 3b.

### Cancer-type-specific metabolites

**Fig. 4:**
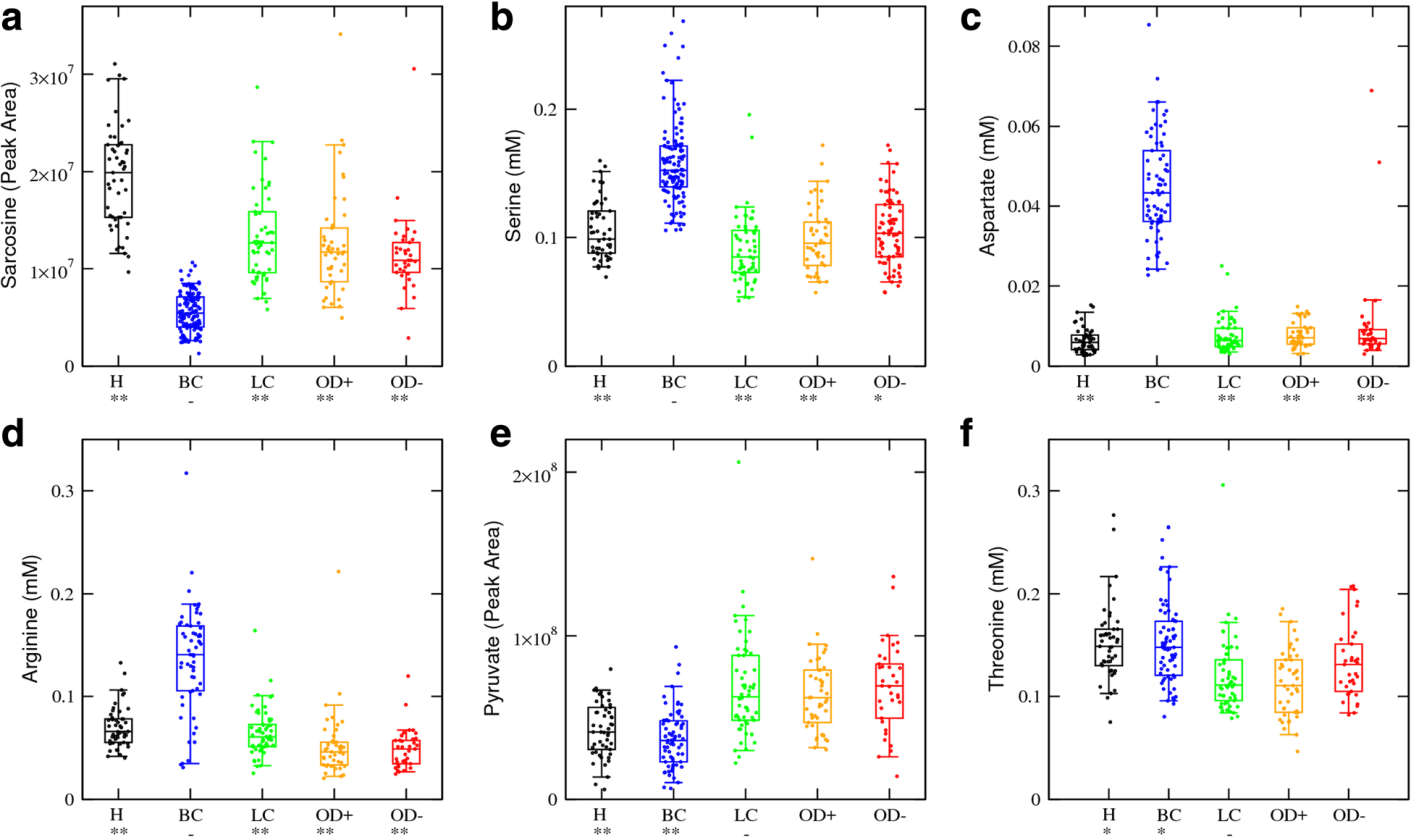
Cancer type specific metabolites. Box plots of metabolite levels manifesting a significant difference between breast or lung cancer and the other groups. The sample groups include healthy controls (H), HER2+ breast cancer (BC), non-small cell lung cancer (LC), severe obesity without diabetes (OD−) and severe obesity with diabetes (OD+) samples. Each point represents a sample, the error bars indicate the range excluding the lowest 5% and highest 5% values, boxes the range excluding the lowest 25% and highest 25% values, and the line within the box the median. Asterisk/double asterisks denote a significant difference of 10^−3^/10^−6^ relative to BC (**a**–**d**) or LC (**e**, **f**), two-tailed *t* test with unequal variance

As discussed above, formate, sarcosine and glutamate exhibit differences in the same direction when comparing breast or lung cancer with healthy controls. There are also metabolites showing cancer-type-specific changes (Additional file 1: Table S1). Sarcosine itself is significantly lower in breast cancer than in all other groups, including lung cancer (Fig. 4a). By contrast, serine, aspartate and arginine are significantly higher in breast cancer as compared with all other groups (Fig. 4b–d). In the case of lung cancer, pyruvate is higher and threonine is lower than in the breast cancer, but not significantly different from the obesity samples (Fig. 4e, f). These metabolites could be exploited to further stratify the cancer samples into breast or lung cancer. However, we cannot fully address their relevance within this work. We are lacking a quantification of their levels in other cancer subtypes such as brain, blood, colorectal and ovarian cancers, which have an incidence comparable with that of breast and lung cancers.

## Discussion

This analysis indicates that formate is a promising biomarker for cancer diagnosis. To address the relevance of formate in a wider context, we searched the scientific literature for previous studies measuring circulating formate in clinical samples and healthy controls or in animal models of human disease (Table 2). In most investigations, formate was quantified using nuclear magnetic resonance (NMR), except for one case utilising an enzymatic method. A study in rhesus monkeys reported significantly lower levels of formate in animals with type 2 diabetes than in matched controls [12]. In an investigation of patients with colorectal cancer in Denmark, it was noted that obese patients had significantly lower serum formate levels than non-obese controls [13]. These studies support our observation of lower serum formate levels in obese individuals.

**Table 2:** Studies reporting formate measurements in humans and primates

Study/condition	Country	Sample	Assay	Cases	Controls	Formate	Fold change	Significance	Ref
Death vs asymptomatic/methanol poisoning	Czech Republic	Serum	Enzymatic	6	15	↑	8	4.0E−3	[26]
Amyotrophic lateral sclerosis	India	Serum	NMR	30	25	↑	NR	< 0.001	[24]
Parkinson’s disease vs healthy	India	Serum	NMR	17	22	↑	3	< 0.001	[25]
Crohn’s disease	Canada	Serum	NMR	20	40	↑	NR	< 0.05	[23]
Ulcerative colitis	Canada	Serum	NMR	20	40	↑	NR	< 0.05	[23]
Non-Hodgkin’s lymphoma	Poland	Serum	NMR	26	31	↑	1.7	< 0.05	[18]
Chronic lymphocytic leukaemia	Poland	Serum	NMR	21	31	↑	1.5	< 0.05	[18]
Acute myeloid leukaemia	Poland	Serum	NMR	38	31	↑	2.0	< 0.05	[18]
Colorectal cancer vs healthy	China	Other^a^	NMR	127	43	↑	NR	1.0E−3	[17]
Colorectal cancer vs healthy	Denmark	Serum	NMR	153	139	↑	1.2	1.5E−5	[13]
Colorectal cancer vs healthy	China	Serum	NMR	28	55	–	NR	> 0.05	[27]
Chronic pancreatitis vs healthy	China	Plasma	NMR	20	20	↑	NR	< 0.05	[28]
Pancreatic cancer vs healthy	China	Plasma	NMR	19	20	–	NR	> 0.05	[28]
Hepatocellular carcinoma	European	Serum	NMR	114	222	–	NR	> 0.05	[29]
Hepatocellular carcinoma	China	Serum	NMR	24	60	–	0.25	> 0.05	[30]
Hepatocellular carcinoma	China	Serum	NMR	43	18	↓	0.07	< 0.05	[16]
Liver cirrhosis	China	Serum	NMR	42	18	↓	0.36	< 0.05	[16]
Hepatocellular carcinoma vs liver cirrhosis	China	Serum	NMR	43	42	↓	0.20	< 0.001	[16]
Lung cancer vs healthy	China	Serum	NMR	39	43	–	0.87	> 0.05	[31]
Lung cancer vs healthy	Portugal	Plasma	NMR	85	78	↓	0.7	3.6E−5	[14]
Lung cancer vs healthy	China	Serum	NMR	27	24	↓	0.5	NA	[15]
Progressive disease vs response/breast cancer	Singapore	Serum	NMR	7	22	↓	NR	< 0.05	[32]
Obesity vs Non/colorectal cancer	Denmark	Serum	NMR	78	21	↓	NR	7.8E−3	[13]
Type 2 diabetes vs healthy	Other^b^	Serum	NMR	8	8	↓	0.3	< 0.05	[12]

^a^Tumour/normal mucosa

^b^Rhesus monkeys

In the context of cancer, we found reports of both low or high serum formate depending on the cancer type. Patients with lung cancer [14, 15] or hepatocellular carcinoma [16] are characterised by lower serum levels of formate, whereas formate is found significantly higher in the serum of patients with colorectal [13, 17] and blood [18] cancer. It seems there is a subset of cancers with lower serum formate levels than in healthy controls (e.g. lung and breast) and another subset of cancers where serum formate levels are higher than in healthy controls (e.g. colorectal). These observations are in line with the current view that different cancer types may represent different metabolic phenotypes.

It is unclear why there is a dichotomy of formate levels in cancer relative to healthy controls. Interestingly, those cancers exhibiting lower serum formate (breast and lung) are among those where increased cell proliferation is a marker of poor prognosis. By contrast, colorectal cancer, showing high serum formate, is among those where increased tissue remodelling is a marker of poor prognosis [19]. Increased cell proliferation and tumour growth could drive the depletion of endogenous sources of one-carbon units, which sustain the biosynthesis of nucleotides [20, 21]. The oxidative or reductive nature of the cancer could be a relevant factor. Data from mouse models indicate that cancers with high oxidative metabolism are associated with increased serum levels of formate [6]. However, we observed low formate levels in the serum of human lung cancer patients, and human lung cancer can be of oxidative nature [22]. Other factors such as alterations in liver function, the immune system and the gut microbiome cannot be excluded.

Finally, we found reports of increased serum formate levels in patients with inflammatory diseases (ulcerative colitis and Crohn’s disease [23]) and neurological diseases (amyotrophic lateral sclerosis [24] and Parkinson’s disease [25]) relative to healthy controls. Thus, the use of serum formate as a potential biomarker for cancers with high serum formate will need further consideration. Indeed, we would need additional biomarkers to discriminate between high serum formate cancer, amyotrophic lateral sclerosis and Parkinson’s disease. The latter findings further emphasise the need to collect samples from multi-disease cohorts and the requirement of multinomial classifiers to impute the different classes based on serum metabolomics or other biomarkers.

## Conclusions

We conclude that circulating formate levels are significantly lower in breast cancer, lung cancer and highly obese patients than in healthy controls. The circulating formate levels together with those of glucose can be used to stratify cancer patients, obese individuals and healthy controls. Further studies are required to determine the relevance of these observations in the context of other human diseases and early diagnosis.

## Additional file

Additional file 1: **Table S1.** Candidate cancer biomarkers. **Table S2.** Comparisons of amino acid concentrations reported in human plasma and present in the amino acid mixture. **Figure S1.** Formate quantification quality controls. **Figure S2.** Quantified amino acid concentrations in the human plasma samples. **Figure S3.** Peak areas (extracted ion counts) for some of the heavy labelled amino acids used as internal standards. **Figure S4.** Relative peak areas of the internal standards in breast cancer samples compared with other samples. Figure S5. Peak areas of EDTA and citrate. (PDF 820 kb)

## Abbreviations

Acot: Cis aconitate
Ala: Alanine
Arg: Arginine
Asn: Asparagine
Asp: Aspartate
BMI: Body mass index
Cit: Citric acid
Creat: Creatine
Ctr: Citrulline
For: Formate
FPR: False positive rate
Fum: Fumarate
GC-MS: Gas chromatography–mass spectrometry
Gluc: Glucose
Gly: Glycine
Gua-Ac: Guanidinoacetate
Lac: Lactate
LC-MS: Liquid chromatography–mass spectrometry
NAFLD: Non-alcoholic fatty liver disease
NASH: Non-alcoholic steatohepatitis
NMR: Nuclear magnetic resonance
pCreat: Creatine phosphate
Phe: Phenylalanine
Pro: Proline
Pyr: Pyruvic acid
ROC: Receiver operating characteristic
Sarc: Sarcosine
Ser: Serine
Succ: Succinate
T2DM: Type 2 diabetes mellitus
Thr: Threonine
TPR: True positive rate
Trp: Tryptophan
Ura: Urate
