# Supplementary material for: Stratification of cancer and diabetes based on circulating levels of formate and glucose
Source: Cancer Metab. 2019 Apr 24;7:3. doi: 10.1186/s40170-019-0195-x (PMC6482583; doi:10.1186/s40170-019-0195-x)
Supplement: Supplementary file 1 — Table S1. Candidate cancer biomarkers. Table S2. Comparisons of amino acid concentrations reported in human plasma and present in the amino acid mixture. Figure S1. Formate quantification quality controls. Figure S2. Quantified amino acid concentrations in the human plasma samples. Figure S3. Peak areas (extracted ion counts) for some of the heavy labelled amino acids used as internal standards. Figure S4. Relative peak areas of the internal standards in breast cancer samples compared with other samples. Figure S5. Peak areas of EDTA and citrate. (PDF 820 kb) [file 40170_2019_195_MOESM1_ESM.pdf]

SUPPLEMENTARY INFORMATION

Multinomial diagnosis of cancer and diabetes using serum levels of glucose and formate

Matthias Pietzke[1,*], Salvador Fernandez Arroyo[2,*], David Sumpton[1], Gillian M Mackay[1], Begoña Martin-Castillo[3**], Jordi Camps[2], Jorge Joven[2], Javier A. Menendez[4,5**], Alexei Vazquez[1,6,†]

[1]Cancer Research UK Beatson Institute, Glasgow, UK
[2]Unitat de Recerca Biomèdica, Hospital Universitari de Sant Joan, IISPV, Rovira i Virgili University, Reus, Spain
[3]Unit of Clinical Research, Catalan Institute of Oncology, Girona, Spain
[4]ProCURE (Program Against Cancer Therapeutic Resistance), Metabolism & Cancer Group, Catalan Institute of Oncology, Girona, Catalonia, Spain
[5]Girona Biomedical Research Institute (IDIBGI), Girona, Spain
[6]Institute of Cancer Sciences, University of Glasgow, Glasgow, UK

* These authors contributed equally to this work
** On behalf of the METTEN study group (EudraClinicalTrial Number 2011[illegible]000490[illegible]30).

Section	Page
**Supplementary Tables**	**1-3**
**Supplementary Figures**	**4-8**
**Amino acid quantification for LC-MS quality control**	**9**
**Calculation of the FPR in multi-disease cohorts**	**10**

	Breast Cancer (BC)		Lung Cancer)LC)		
**Metabolite**	**Fold change**	**Significance**	**Fold change**	**Significance**	**Putative Cancer Biomarker**
Alanine	0.13	0.092048	-0.16	0.098497	
Arginine	0.95	0.000000	-0.12	0.180788	
Asparagine	0.11	0.054046	-0.22	0.001387	
Aspartate	2.79	0.000000	0.24	0.110279	
Glutamate	0.21	0.016518	0.24	0.005497	YES
Glutamine	-0.64	0.006407	0.10	0.594331	
Glycine	0.51	0.000000	-0.14	0.061204	
Isoleucine	-0.06	0.457252	-0.18	0.024684	
Leucine	-0.05	0.505865	-0.26	0.001420	
Lysine	0.04	0.500180	-0.18	0.001562	
Methionine	-0.13	0.251328	-0.35	0.006930	
Phenylalanine	0.39	0.000000	-0.06	0.320079	
Proline	0.07	0.381282	0.33	0.000637	
Serine	0.59	0.000000	-0.22	0.002411	
Threonine	0.00	0.964455	-0.33	0.000025	
Tryptophan	-0.05	0.308610	-0.24	0.000173	
Tyrosine	-0.01	0.833279	-0.06	0.377414	
Valine	-0.04	0.467981	-0.17	0.003204	
Formate	-0.90	0.000000	-0.69	0.000000	YES
cis_Aconitate	-1.02	0.000000	0.30	0.000968	
Citric_acid	-0.45	0.000001	0.03	0.708656	
Citrulline	-0.16	0.067902	-0.16	0.088824	
Creatine	0.63	0.000043	-0.41	0.025010	
Creatine_phosphate	0.00	1.000000	-0.40	0.000336	
Creatinine	-0.19	0.001646	0.12	0.113318	
Cystathionine	0.38	0.264843	0.68	0.030700	
Fumarate	-0.62	0.000002	0.18	0.107540	
Glucose	0.00	0.986658	0.23	0.056850	
Guanidinoacetate	-0.02	0.770685	-0.20	0.047359	
Lactate	-0.32	0.000002	0.06	0.446430	
Malate	-0.31	0.001353	0.23	0.013745	
Ornithine	-0.27	0.014182	-0.13	0.197653	
Pyruvic_acid	-0.20	0.084038	0.67	0.000001	
Sarcosine	-1.91	0.000000	-0.41	0.000686	YES
Succinic_acid	-0.81	0.000010	0.01	0.961266	
Urate	-0.06	0.304477	0.13	0.023692	

Table S1. Candidate cancer biomarkers. Fold change and statistical significance of quantified metabolite levels in cancer patients relative to healthy controls. Statistical significance was determined using the two-tailed Welch test. The cancer biomarker column indicates metabolites that are significantly and consistently lower or higher in both breast and lung cancer patients relative to healthy controls.

Metabolite	Reported concentration in plasma [1] (µM)	Expected concentration in LC-MS preparation (µM)	Concentration of internal standard in extract (mM)	concentration of standard in LC-MS preparation(µM)	Ratio Sample / IS
Alanine	430	4.3	73	3.65	1.18
Arginine	114	1.14	24	1.20	0.95
Asparagine	82	0.82	19	0.95	0.86
Aspartate	21	0.21	58	2.90	0.07
Cysteine	34	0.34	20	1.00	0.34
Glutamine	510	5.1	20	1.00	5.10
Glutamate	97	0.97	47	2.35	0.41
Glycine	325	3.25	54	2.70	1.20
Histidine	131	1.31	5	0.25	5.24
Isoleucine	61	0.61	24	1.20	0.51
Leucine	98.7	0.987	51	2.55	0.39
Lysine	179	1.79	21	1.05	1.70
Methionine	30	0.3	10	0.50	0.60
Phenylalanine	78	0.78	19	0.95	0.82
Proline	198	1.98	21	1.05	1.89
Serine	160	1.6	31	1.55	1.03
Threonine	128	1.28	32	1.60	0.80
Tryptophan	55	0.55	20	1.00	0.55
Tyrosine	55	0.55	15	0.75	0.73
Valine	212	2.12	32	1.60	1.33

Table S2. Comparisons of amino acid concentrations reported in human plasma and present in the amino acid mixture. The expected concentrations as injected into the LC-MS are also reported, following a 1:100 dilution for samples and a 1:20.000 dilution of the standards in the extraction solvent.

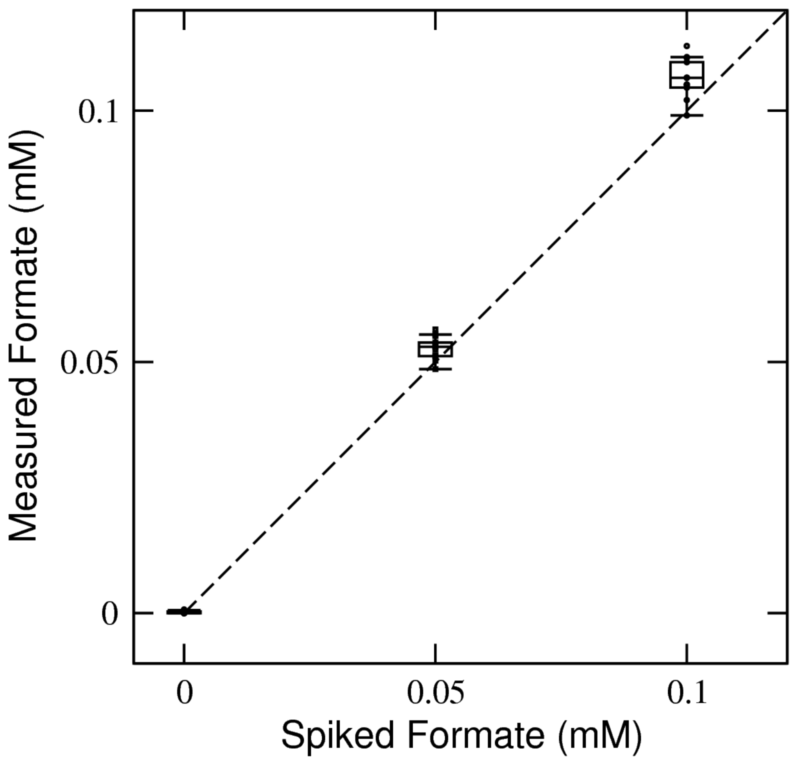

Figure S1. Formate quantification quality controls. In each batch of samples subject to formate quantification, we added 3 reference samples of 0, 0.05 and 0.1 mM formate. The plot reports the measured formate levels for these three samples across all batches. The dashed line indicates a perfect agreement between the formate quantification and the expected value.

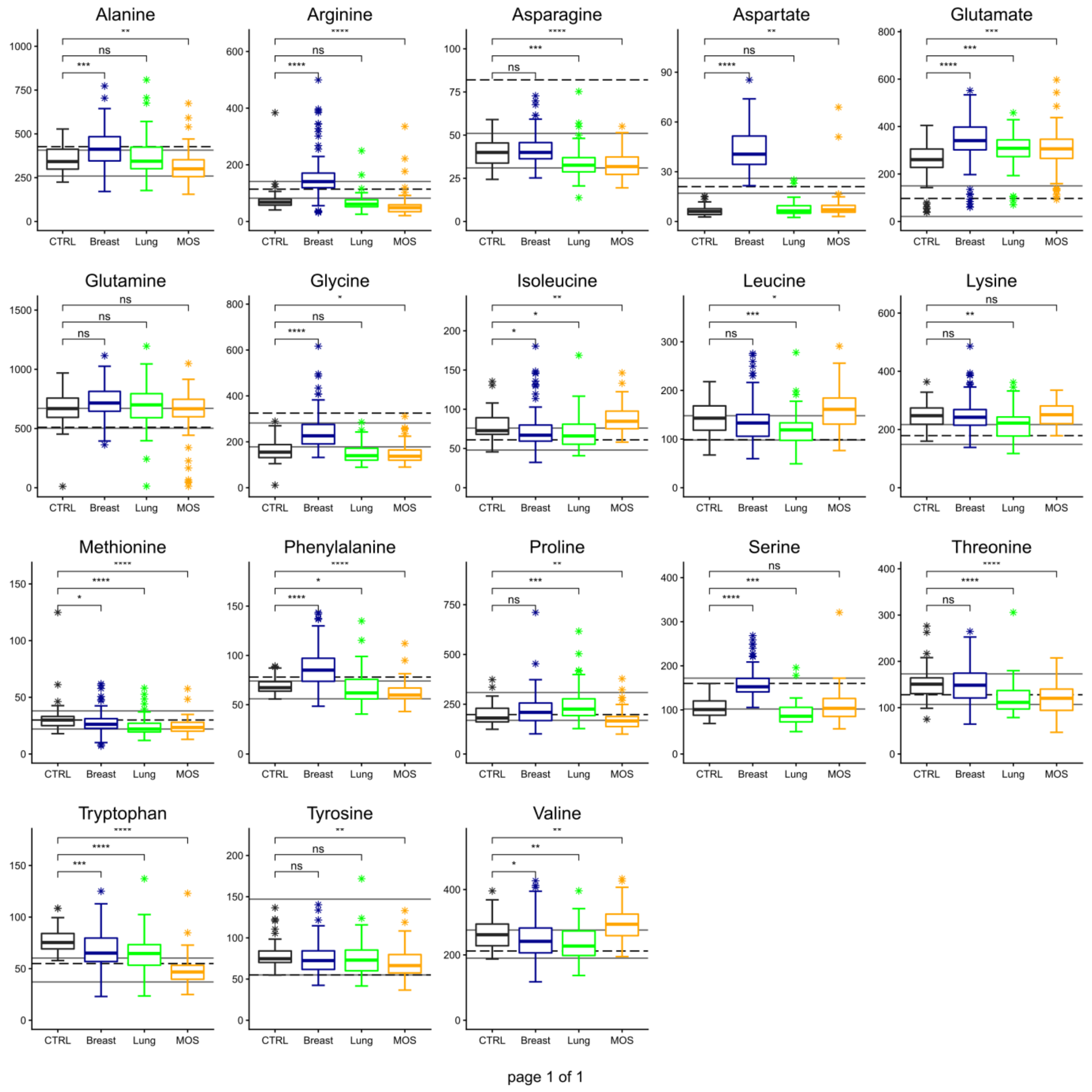

Figure S2. Quantified amino acid concentrations in the human plasma samples. All concentrations are expressed as μM. The black dashed line indicates the concentration reported in [21] and the grey solid lines indicate the min and max concentration referenced therein.

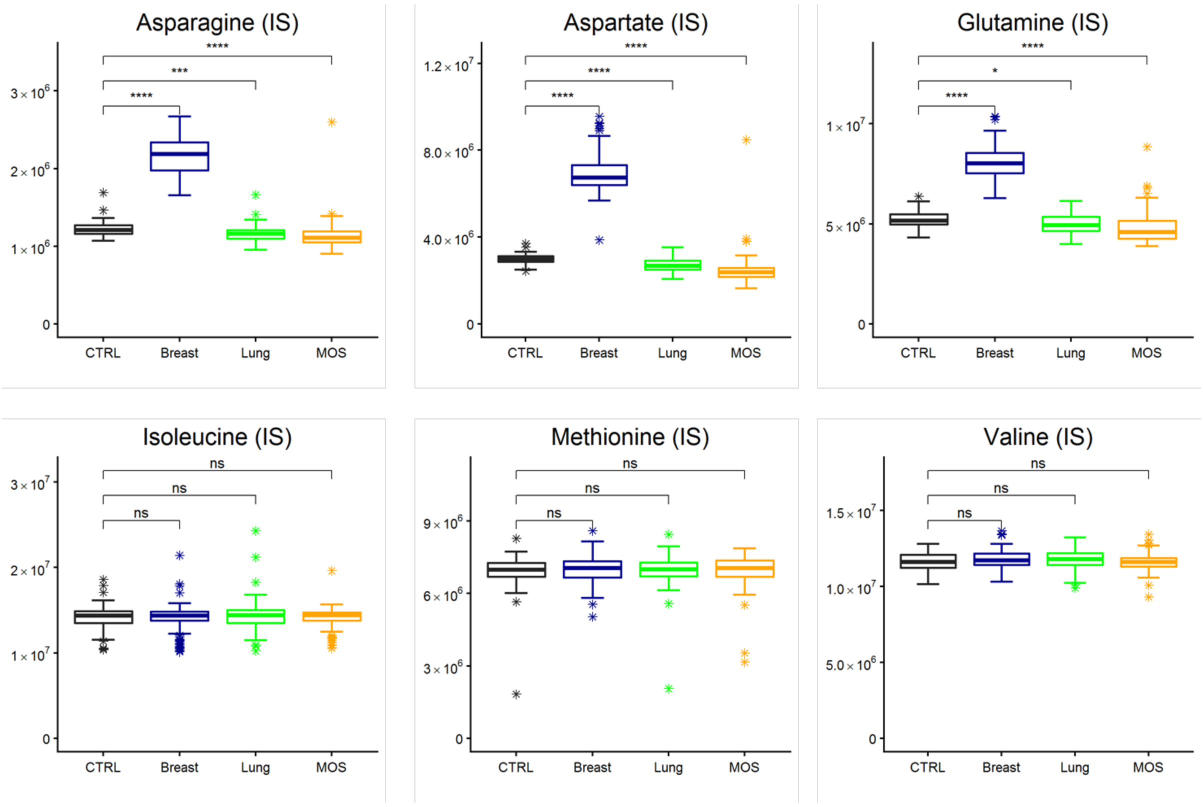

Figure S3. Peak areas (extracted ion counts) for some of the heavy labelled amino acids used as internal standards. Some of the compounds such as glutamine, aspartate and asparagine show extremely high values in the breast cancer samples (top row), while others are measured consistently across the samples, as would be expected (bottom row).

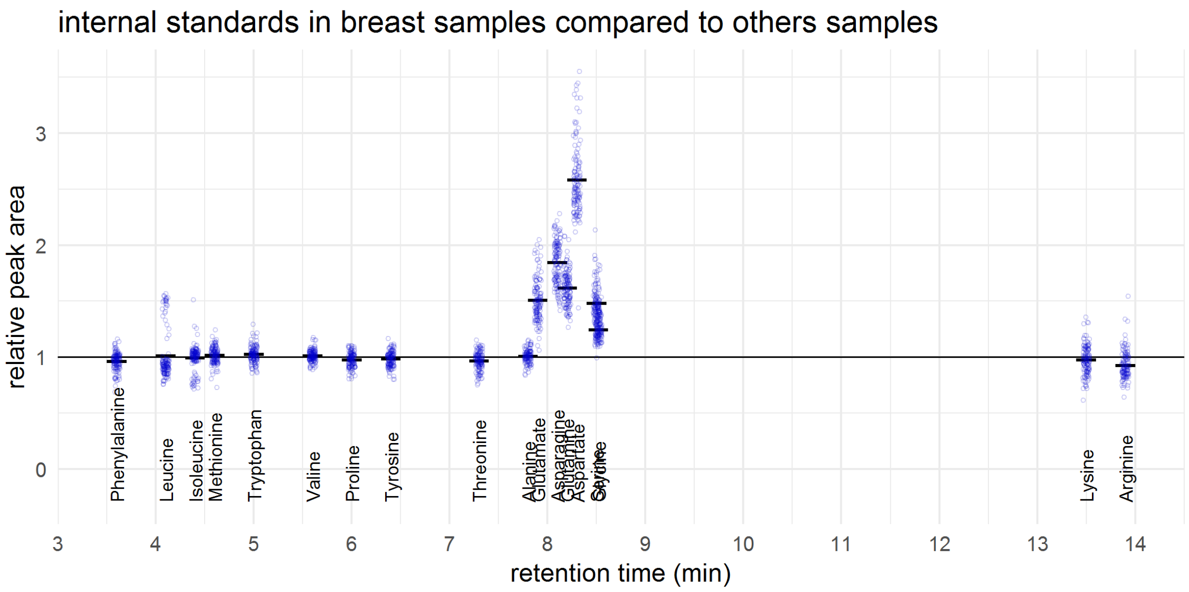

Figure S4. Relative peak areas of the internal standards in breast cancer samples compared with other samples. Fold change was calculated for the breast cancer samples relative to the average of all the other samples. Each dot represents a measurement and the short black lines the average of all measurements per compound. The amino acids are indicated with their name.

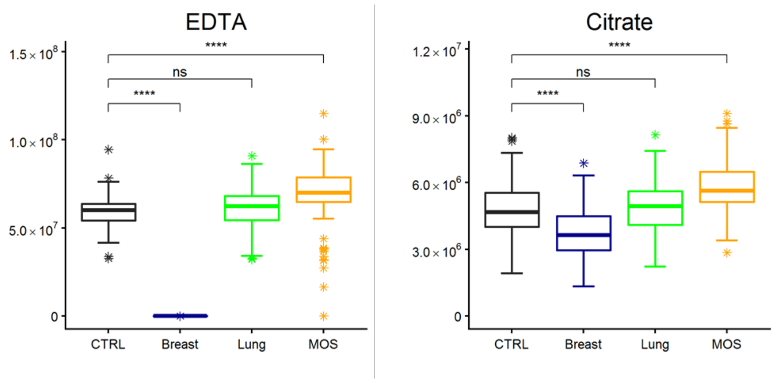

Figure S5. Peak areas of EDTA and citrate. EDTA tubes were used for the collection of all samples except for the breast cancer samples. Also citrate-tubes can be excluded for the breast cancer sample collection; its intensity is similar between the samples and reflects biological variations between the sample types.

Amino acid quantification for LC-MS quality control

For the quantification of amino acids in either plasma or serum, we extracted the samples in an extraction solvent spiked with a u-^{13}C,^{15}N cell-free amino acid mixture (Sigma-Aldrich) at a dilution of 1:20,000. Although the relative proportion of each amino acid in the standard mixture is different to the proportion in plasma or serum, the majority of amino acids are present in a similar concentration as expected[21]. Only glutamine and histidine are more abundant in the plasma (appr. 5×), whereas aspartate is 14× more abundant in the standard and cysteine, glutamate and leucine are 3× more abundant in the standard.

Using this approach, we were able to quantify 18 of the 20-proteinogenic amino acids (**Table S2**, **Figure S2**). Only histidine and cysteine were not measured by our method. The detected quantities for most of the compounds are in the concentration range reported previously, demonstrating the validity of this approach. Moreover the concentrations of the amino acids are quite stable between the different sample types.

Matrix effects. We observed some unexpected behaviour for some of the amino acids in some samples. Whereas all samples were extracted using the same extraction solvent, and therefore the spiked-in heavy amino acids were present at a uniform concentration, there were significant differences between the breast-cancer samples and the other sample types, as shown for asparagine, aspartate and glutamine (**Figure S3**, *top row*). Oddly, this was found for only a few amino acids, as others were measured similarly across all samples (e.g. isoleucine, methionine, valine, **Figure S3**, *bottom row*). Closer inspection of this phenomenon revealed that all affected amino acids eluted in the same retention time window, between 8.0 and 8.5 minutes (**Figure S4**). Moreover, we found that EDTA also eluted in this retention time window, in all samples but the breast cancer samples. The abundance of EDTA in the samples results in signal repression for the bulk of samples except for breast cancer samples, where EDTA is absent and therefore the signal is much stronger (**Figure S5**). As the amino acids and the spiked-in internal standard should be affected in the same way, normalising the amino acid with help of the internal standard should correct for this effect. However compounds eluting in this retention time window should be evaluated with greater care.

Calculation of the false positive rate (FPR) in multi-disease cohorts

The calculation of FPR in multi-disease cohorts is based on the following reasoning. Suppose we have a cohort with a set of samples *U*, divided into a set *H* of healthy controls and sets of diseases D_i (i=1,…,n) where n is the number of diseases in the cohort. In addition we have an estimate of the prevalence p_i (i=1,…,n) of these different diseases in the human population. Suppose the samples are also assigned to a set H^* of healthy controls and sets of diseases D_i^* (i=1,…,n) based on some classifier. Under the assumption that co-morbidities are negligible, the FPR associated with the classification of disease i is given by the equation

$$TPR_i = \frac{|V \cap H \cap D_i^*| + \sum_{j \neq i} x_j |V \cap D_j \cap D_i^*|}{|V \cap H| + \sum_{j \neq i} x_j |V \cap D_j|}$$

$$x_j = p_j \frac{|V \cap H|}{|V \cap D_j|}$$

where V is the validation set of samples, the symbol $|X|$ denotes the size of set X (number of samples in X), and the symbol $X \cap Y$ indicates the union between X and Y (number of common samples between X and Y). We note that the factor x_j assures that each disease contributes in proportion to its prevalence in the population, independently of the relative sizes of the healthy and disease sets in the cohort.
